# Isolation and characterization of a novel highly efficient bacterium *Lysinibacillus boronitolerans* QD4 for quantum dot biosynthesis

**DOI:** 10.3389/fmicb.2025.1521632

**Published:** 2025-01-29

**Authors:** Xingyu Gu, Xiaoju Li, Ruijia Zhang, Ruoli Zheng, Mingrui Li, Rong Huang, Xin Pang

**Affiliations:** ^1^School of Life Sciences, Shandong University, Qingdao, China; ^2^State Key Laboratory of Microbial Technology, Shandong University, Qingdao, China

**Keywords:** *Lysinibacillus boronitolerans*, quantum dot, nanoparticle biosynthesis, cadmium sulfide, extracellular

## Abstract

Microorganism-based biosynthesis of quantum dots is a low-cost and green production method with a wide range of potential applications. The development of environmentally friendly synthesis methods is required due to the toxicity and severe reactions that occur during the chemical synthesis of quantum dots. In this study, a novel strain, QD4, with the ability to the effectively and rapidly biosynthesize CdS quantum dots, is isolated and reported. The isolated strain is a Gram-positive, aerobic, flagellated, and rod-shaped bacterium, isolated from seawater. Through the physio-biochemical characterization and 16S rRNA-based phylogenetic tree analysis, the strain is identified as *Lysinibacillus boronitolerans* QD4. The strain QD4 grows well in the range of 25–40 °C (optimum, 37 °C), pH 5.0–9.0 (optimum, pH 7.0), with a high cadmium-resistance as it could grow at Cd^2+^ concentration up to 2 mM, implying its good adaptability to the environment and potential for application. Cd^2+^ and L-cysteine are used as substrates for the biosynthesis of CdS quantum dots by strain QD4. The distinctive yellow fluorescence from CdS quantum dots is visible after only a short induction time (a few hours). Moreover, the properties of the CdS quantum dots are characterized by fluorescence spectroscopy, UV-absorption spectroscopy, TEM, XRD, XPS, and infrared spectroscopy. This study provides a novel strain resource for efficient biosynthesis of extracellular, water-soluble quantum dots, paving potential industrial applications in green production.

## 1 Introduction

Quantum dots are special nanomaterials. Due to their small size, the physiochemical characteristics of quantum dots are notably different from bulk materials. Cadmium sulfide (CdS), a direct semiconductor with a wide-bandgap (band gap energy of 2.4 eV), is one significant quantum dot. The size-dependent optical properties of CdS quantum dots, such as their tunable light emission, make them useful in a variety of applications, including catalysis, sensors, clinical diagnosis, environmental management, and many other fields (Qiang et al., 2022; Niu et al., 2013; Hu et al., 2014; Dang et al., 2018). Consequently, quantum dots are crucial for many nanotechnology applications. Mostly, quantum dots are synthesized by chemical and physical methods, which usually require toxic reagents and harsh reaction conditions and lead to the generation of toxic by-products (Bao et al., 2010; Xiao et al., 2010). These limitations can be efficiently resolved by synthesizing quantum dots using biological systems. Biosynthesis and purification of quantum dots can be carried out in biological resources using non-toxic reagents without any harsh reaction conditions. As a result, it is a clean, inexpensive, environment-friendly, and biocompatible method for the synthesis of quantum dots.

Various organisms, such as yeast, algae, fungi, bacteria, and plant extracts, have been explored for the biosynthesis of quantum dots (Jacob et al., 2016; Zhu et al., 2018; Du et al., 2017; Vyas et al., 2022). Among these organisms, bacteria are increasingly attracting the research interest for quantum dot biosynthesis due to their ability to grow more quickly and easily than other organisms such as yeast and fungi, even in simple growth media. Furthermore, bacteria can swiftly adapt to the fluctuations in the environment, suggesting a great potential to be utilized as nano-factories for quantum dot synthesis.

One of the most common bacteria, *Escherichia coli* (*E. coli*), has been explored for the biosynthesis of quantum dots due to its rapid growth under simple growth conditions. Therefore, it has been extensively used as a model microorganism to investigate the biosynthesis of nanoparticles. It offers an economical way to produce functional nanoparticles. The biosynthesis of CdS quantum dots has been successfully carried out through recombinant *E. coli*, produced by overexpressing the foreign genes, such as *γ-glutamylcysteine synthetase gene*, *cysteine desulfhydrase gene* of *Treponema denticola*, or *PCS gene* of *Schizosaccharomyces pombe* (Choi et al., 2018; Park et al., 2010; Dingkun et al., 2019; Marusak et al., 2016). Mostly, the nanoparticles are produced intracellularly by *E. coli*, with more than 12 h needed to obtain CdS quantum dots (Tian et al., 2019). The location of nanoparticle biosynthesis by microorganisms needs significant attention. If the nanoparticles produced by bacteria nucleated and grown in the interior of the cell (cytoplasmic, or periplasmic), the harvesting of nanoparticles would be complex, like to require lysing cells. Moreover, the produced intracellular nanoparticles may stick to the intracellular constituents of the cell, such as the cytoplasm or the membrane. This makes it challenging to get independently distributed nanoparticles in the cell lysate. Comparatively, the synthesized extracellular nanoparticles can be easily separated from bacterial cells by centrifugation, remaining most of the nanoparticles dispersed independently in the supernatant. Thus, it is important to develop pathways for the synthesis of nanoparticles that would enable *E. coli* to produce them extracellularly. In addition, previous studies have reported that the minimal inhibitory concentration of Cd against *E. coli* was only 0.4 mM (Mi et al., 2011). Therefore, the toxic effects of Cd on microorganisms need to be considered.

Researchers have also investigated many different bacteria for the biosynthesis of quantum dots in addition to *E. coli*. Likewise, metal- and sulfur-reducing bacteria were explored by some researchers, which showed promising potential for the fabrication of various nanoparticles. CdS quantum dot produced by these bacteria exhibited high degradation of diazo dye, trypan blue (Jang et al., 2015; Qi et al., 2019; Rajput et al., 2021; Chellamuthu et al., 2019). However, these bacteria require anaerobic cultivation, indicating difficult cultivation and slow growth, which is unfavorable for practical applications. Some aerobic bacteria, such as *Pseudomonas putida*, *Acidithiobacillus* sp., *Idiomarina* sp., *Polyextremophile halophilic* sp., and others, have been reported for the biosynthesis of CdS quantum dots. A relatively long time (several days) is needed for the biosynthesis of CdS quantum dots, particularly using certain extremophile microbes (Oliva-Arancibia et al., 2017; Ulloa et al., 2016; Ma et al., 2021; Bruna et al., 2019). The production of CdS quantum dots has also been investigated using a cell-free extract of *P. chlororaphis* CHR05; however, it requires more than 24 h of incubation (Ashengropha et al., 2020).

Therefore, rapid biosynthesis of extracellular, water-soluble CdS quantum dots using microorganisms is important for practical industry applications. To be best of our knowledge, only three bacteria have been reported for very quick (<3 h) and extracellular biosynthesis of water-soluble CdS quantum dots. One is *Stenotrophomonas maltophilia* (SMCD1) strain isolated using the soil collected from the mountaintop. It is a non-fermentative, aerobic, Gram-negative bacterium, which is highly prevalent in the environment and has a high tolerance for Cd^2+^ up to 2 mM (Pages et al., 2008). It can synthesize water-soluble CdS quantum dots extracellularly (Yang et al., 2015). The other bacterium is *Raoultella* sp. X13 strain isolated by Xu et al. from heavy metal-contaminated soil. It is a Gram-positive, aerobic, cadmium-resistant strain that could produce CdS quantum dots using Cd^2+^ and L-cysteine (Xu et al., 2019; Xu et al., 2021). The third one is *Pseudomonas fragi* GC01 strain, which can not only intracellularly biosynthesize CdS quantum dot at low temperatures (15 °C) but can also biosynthesize it extracellularly at normal temperature (28 °C) in the presence of Cd^2+^ and cysteine (Gallardo et al., 2014; Gallardo-Benavente et al., 2019). Particularly, the enzyme from the SMCD1 strain, named cystathionine *γ*-lyases, has been extensively investigated for its capability to synthesize aqueous CdS quantum dots directly from Cd^2+^ and L-cysteine (Dunleavy et al., 2016; Wang et al., 2021; Niu et al., 2024), which can effectively biomineralize CdS nanocrystals with regulated optical properties.

In this research study, a novel bacterium is isolated from seawater and screened for its ability to biosynthesize CdS quantum dots. This bacterium can efficiently and rapidly biosynthesize CdS quantum dots extracellularly. The bacterium is identified as *Lysinibacillus boronitolerans* QD4 through 16S rRNA sequencing, and morphological and physiochemical analysis. The conditions for the bacterial production of CdS quantum dots are systematically optimized. The biosynthesis of quantum dots needs to be performed in the presence of CdCl_2_ and L-cysteine as sulfur sources. The properties of the biosynthesized CdS quantum dots are also analyzed by microscopic and spectroscopic techniques. The study provides a novel strain for the rapid and extracellular biosynthesis of CdS quantum dots, enabling the purification process simpler. The CdS quantum dots biosynthesized by the bacterium with obviously yellow fluorescence need only a few hours. Furthermore, the bacterium shows a good degree of environmental adaptation and a relatively high resistance to Cd. It is very simple to grow in the LB medium, reaching the logarithmic phase less than 10 h. The study opens one possible way for environmentally friendly, low-cost synthesis of CdS quantum dots for further industrial applications.

## 2 Materials and methods

### 2.1 Sample collection

The samples were collected from the seawater (Qingdao Blue Silicon Valley Coastal Park, Qingdao, China). The samples were placed in sterilized plastic bags and transferred directly to the laboratory for further experiments.

### 2.2 Screening of quantum dot synthesizing bacteria

Quantum dot synthesizing bacteria were isolated from seawater samples, through enrichment culture using LB medium. First, seawater samples were inoculated into the LB liquid medium and cultured at 180 r/min at 37 °C for 24 h. Following that, the enriched bacterial solution was seeded on LB agar plates containing a selective medium with CdCl_2_ and cultured at 37 °C. Single colonies were picked for isolation, purification, and microscopic analysis. In this way, the purified cadmium-resistant bacterial strains were obtained.

The purified Cd-tolerant strains were inoculated in the LB liquid medium. After 24 h of incubation at 180 r/min and 37 °C, 1 mM CdCl_2_ and 8 mM L-cysteine were added to induce the synthesis of quantum dots. At 2-h intervals, 1 mL of sample was taken and centrifuged at 12000 rpm for 5 min. The fluorescence luminescence of the samples was observed and recorded by a UV lamp at 365 nm. Controls were set up without CdCl_2_ and L-cysteine. A significant difference between the fluorescence luminescence of the sample and control indicated the possible generation of CdS quantum dots. The absorbance and fluorescence emission spectra of supernatants were measured using an ultraviolet–visible spectrophotometer (UV-3000, Japan) and a fluorescence photometer (F-7000, Japan), at 365 nm excitation wavelength using a 5-nm excitation slit width.

### 2.3 Identification of the isolated strains

The selected strains were identified through colony morphology, Gram staining, bacterial morphology, physio-biochemical analysis, 16S rRNA sequencing, and phylogenetic tree construction. Physiological and biochemical reaction tubes were procured from Qingdao HaiBo Bio-Tech Co., Ltd. Furthermore, 16S rRNA was sequenced at Qingdao RuiBiotech Co., and the phylogenetic tree was constructed using MEGA-X software (Kumar et al., 2018).

### 2.4 Evaluation of the biosynthesis of CdS quantum dot by screened bacteria

The capability of selected isolates to produce quantum dots was explored. After 24 h culture in the LB liquid medium at 37 °C, the grown bacterium was collected by centrifugation. Thereafter, six sterile tubes were taken to set up six groups with different compositions: (1) LB medium, bacterium, CdCl_2_ (1 mM) and L-cysteine (8 mM); (2) LB medium, bacterium and L-cysteine (8 mM); (3) LB medium, bacterium and CdCl_2_ (1 mM); (4) sterile water, bacterium, CdCl_2_ (1 mM) and L-cysteine (8 mM); (5) LB medium, CdCl_2_ (1 mM) and L-cysteine (8 mM); and (6) sterile water, CdCl_2_ (1 mM) and L-cysteine (8 mM). The biogenesis of CdS quantum dots was investigated by incubating all six samples at 37 °C and 180 r/min. When the fluorescence emerged, a UV spectrophotometer and fluorescence photometer were used to record the UV absorption and fluorescence spectra, respectively. All groups consisted of samples set of three replicates to verify the experimental reproducibility.

#### 2.4.1 Optimization of growth conditions for quantum dot synthesizing bacteria

Based on screening results, *Lysinibacillus boronitolerans* QD4 was selected for the biosynthesis of CdS quantum dots. The effects of temperature and pH on QD4 strain were evaluated to optimize the growth conditions for the synthesis of quantum dots. After the strain was activated in the LB liquid medium and grew up to the OD _600 nm_ = 1, 1% of the culture was inoculated into the fresh LB liquid medium. To determine the influence of temperature on its growth, the QD4 strain was cultured at 180 r/min at several temperatures: 25 °C, 30 °C, 35 °C, 37 °C, 40 °C, and 45 °C, while maintaining a constant pH of 7. Three parallel replicates were used for each group. Cultures were sampled every 3 h to test the OD _600 nm_. Similarly, the strain was cultured at 180 r/min at a constant temperature (37 °C) to identify the optimal pH, which varied from 3 to 11. Each group had three parallel samples. In every 3 h, culture samples were taken to test the OD _600 nm_.

#### 2.4.2 Determination of optimum Cd^2+^ concentration for quantum dot synthesis

The strain was cultured at pH 7, 37 °C, and 180 r/min with different Cd^2+^ concentrations of 0, 0.5, 1.0, 1.5, 2.0, and 2.5 mmol/L to optimize the Cd^2+^ concentration for quantum dot synthesis. There were three parallel samples in each group. In every 3 h, samples were taken to test the OD _600 nm_.

#### 2.4.3 Selection of appropriate sulfur source

Cysteine and glutathione were used as different sulfur sources to select the appropriate sulfur source for the biosynthesis of quantum dots using the isolated strain (Dunleavy et al., 2016; Li et al., 2024). Both sulfur sources were added to the cultured bacterial solution in the presence of 1 mM Cd^2+^, respectively. The fluorescence luminescence of the samples was observed and recorded by a UV lamp at 365 nm. One important factor in choosing the sulfur source is the induction time required for the typical yellow fluorescence from CdS quantum dots to be observed.

#### 2.4.4 Effect of induction time

Following the addition of Cd^2+^ and L-cysteine to the bacterial solution, samples were taken every 30 min and irradiated by a 365 nm UV lamp. Meanwhile, fluorescence and UV absorption spectra at different times were recorded using the fluorescence spectrophotometer and UV–visible spectrophotometer, respectively.

### 2.5 Characterization of CdS quantum dots

#### 2.5.1 Transmission electron microscopy analysis

Bacterial morphology was observed by transmission electron microscopy (TEM): Cells of *Lysinibacillus boronitolerans* QD4 in the log-growth phase were collected by centrifugation. After being washed, cell pellets were resuspended in 0.1 M phosphate-buffered saline (pH 7.4). A small amount of the cell suspension was applied onto a copper grid coated with amorphous carbon film. Following that, the cells were negatively stained using phosphotungstic acid (pH 6.5) for 1 min. Thereafter, the bacterial morphology was examined using a Tecnai G2 F20 transmission electron microscope, operated at 200 kV accelerating voltage.

Biosynthesized CdS quantum dots were observed by TEM: For this purpose, *Lysinibacillus boronitolerans* QD4 was cultured in the LB liquid medium with the addition of Cd^2+^ and L-cysteine. When the yellow fluorescence was visible, a drop of the cell suspension was taken and deposited on copper TEM grids coated with ultrathin amorphous carbon film. TEM analyses were performed using a Talos F20X transmission electron microscope (Thermo Fisher), operated at 200 kV accelerating voltage, equipped with a field-emission gun and a four-detector Super-X energy-dispersive X-ray spectrometer, and capable of working in both conventional TEM and scanning transmission (STEM) modes. Elemental compositions were determined using energy-dispersive X-ray spectrometry (EDS) in STEM mode.

#### 2.5.2 Macroscopic analysis of CdS quantum dot

After a relatively long reaction time (approximately 24 h), CdS nanoparticles grew and appeared as yellow precipitation in the bacterial solution with Cd^2+^ and L-cysteine. The yellow precipitation was collected, washed, and dried to obtain a powder form for X-ray diffraction (XRD), X-ray photoelectron spectroscopy (XPS), and Fourier-transform infrared (FTIR) spectroscopy. XRD was performed using a Rigaku Smart Lab 9 kW instrument with Cu Kα (1.542 Å) radiation. The obtained diffraction spectra of samples were compared with the standard XRD patterns of CdS (PDF card no. 89–0440) from the International Centre for Diffraction Data (ICDD). XPS analysis was carried out using a Thermo Fisher ESCALAB 250XI X-ray photoelectron spectrometer. FTIR spectra were acquired through a Bruker VERTEX 70v spectrometer with 4 cm^−1^ resolution, at wavenumber ranging from 1,000 to 4,000 cm^−1^.

## 3 Results

### 3.1 Screening and characterization of quantum dot synthesizing bacteria

Twenty-three bacterial strains with high cadmium resistance are isolated from seawater. Among them, only 14 strains could produce yellow fluorescence of CdS quantum dots during the induction process. Three strains synthesized CdS quantum dots more rapidly than the other strains. Based on the strain identification results, two of them are identified to be potentially pathogenic. Therefore, the non-pathogenic QD4 strain is chosen for the subsequent studies based on the maximum biosynthesis efficiency and biological safety.

**Figure 1:**
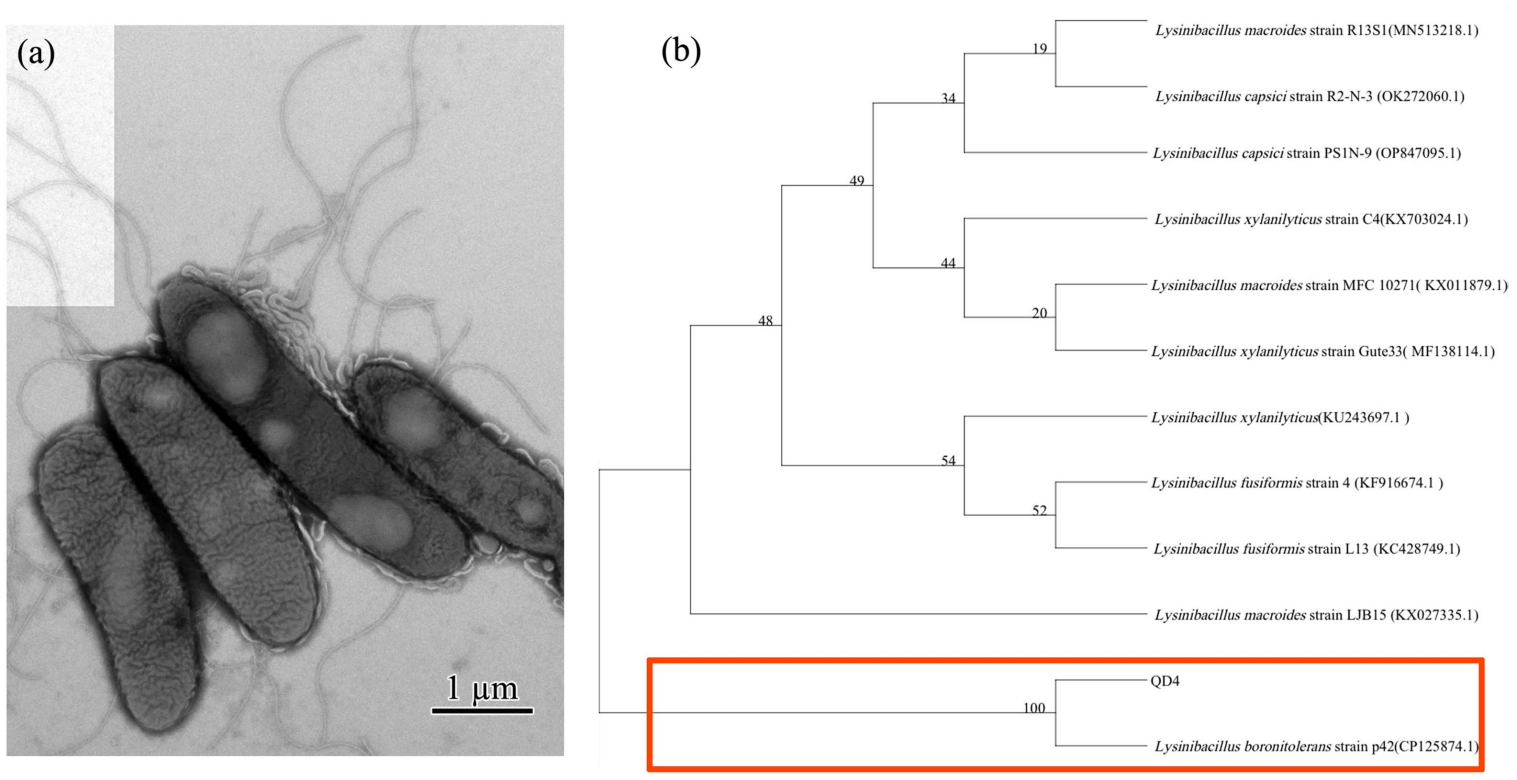
CdS quantum dot synthesizing bacteria of *Lysinibacillus boronitolerans* QD4 isolated from seawater. **(A)** TEM morphology and **(B)** phylogenetic tree based on 16S rRNA sequence.

The morphology of the QD4 bacterial strain is examined by TEM. It is a rod-shaped bacterium with a size of (0.75 ~ 0.9) × (2.48 ~ 4.2) μm, peripheral flagellum, and dense cilia on its surface (Figure 1A). Phylogenetic analysis based on 16S rRNA gene sequence revealed that QD4 shared high similarity (> 99% identity) with *Lysinibacillus boronitolerans* strains in the openly accessible database (Figure 1B). Thus, strain QD4 is verified to be a *Lysinibacillus boronitolerans* QD4. Furthermore, the physio-biochemical analysis of QD4 was carried out, and the results are shown in Table 1.

**Table 1:** Results of physio-biochemical analysis and Gram staining: +, positive; −, negative.

Physio-biochemical reactions	Results	Physio-biochemical reactions	Results
Urea enzymes	+	MR-VP	+
H_2_S	−	Caenorrhodopsin	−
Gelatin liquefaction	−	Glucose produces acid	−
Amylolysis	−	7% NaCl	+
Mannitol	−	3% H_2_O_2_	+
Fructopyranose	+	Dynpower-nitrate culture base	+
Saccharose	−	Glucose oxidase	+
Lactobiose	−	Glyceridase	+
Simoncitrate	+	Gram stain	+
Wood sugar	−		

**Figure 2:**
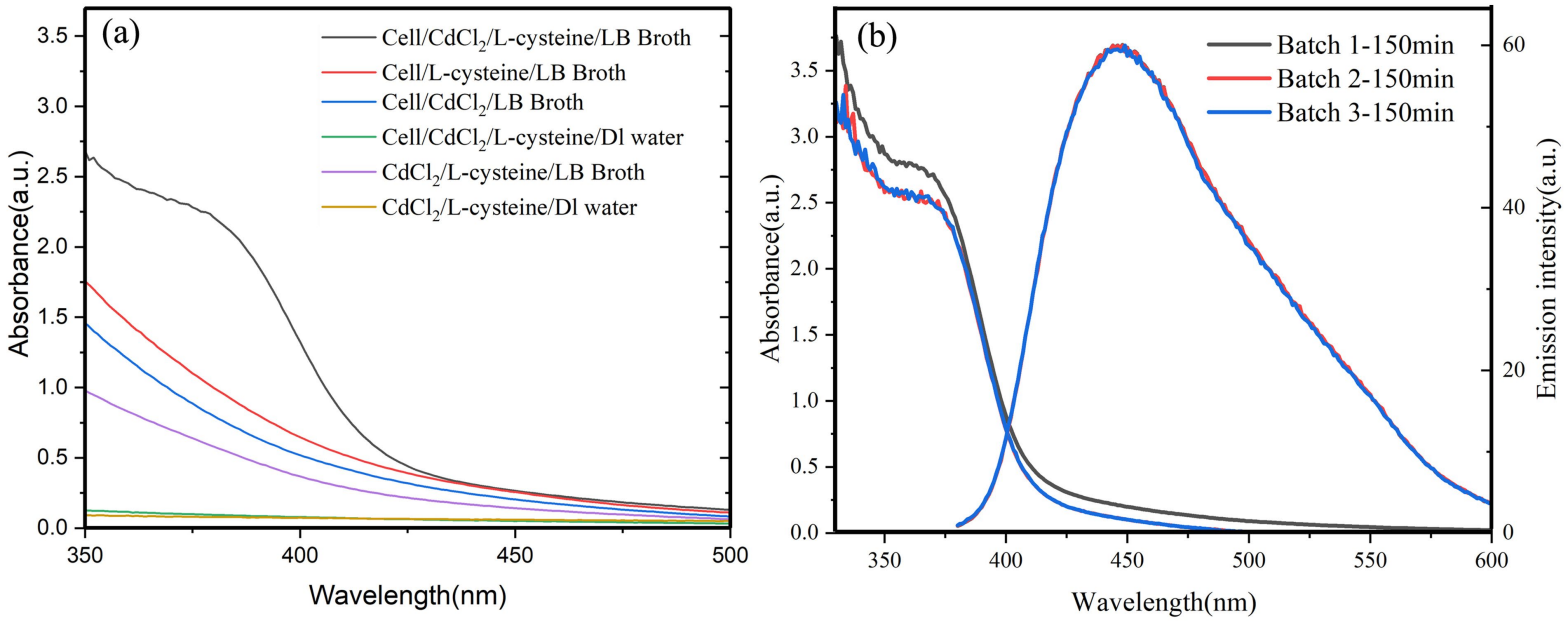
Absorption and emission spectra of QD4 bacterial cultures. **(A)** UV absorption spectra of several comparative samples show that only QD4 cells cultured in the LB media with the presence of both CdCl_2_ and L-cysteine result in the formation of CdS quantum dots. **(B)** Optical characteristics of three different batches of CdS quantum dots prepared using the same incubation conditions show good reproducibility. The emission spectra were recorded using a 365 nm excitation wavelength.

The conditions for the biosynthesis of CdS quantum dots by QD4 are evaluated. The results demonstrate that the absorbance and fluorescence signals corresponding to the CdS quantum dots are observed only when QD4 was cultured in the LB medium in the presence of both Cd^2+^ and L-cysteine for 2–4 h (Figure 2). This indicates the production of CdS quantum dots by QD4. No fluorescence was observed in other groups lacking bacterium strain QD4, Cd^2+^, or L-cysteine. This finding suggests that the isolated strain QD4 could synthesize CdS quantum dots in the presence of Cd^2+^ and L-cysteine. Moreover, the optical characteristics of QD4 were seen in all three parallel samples cultured under optimized growth conditions with the same incubation time, demonstrating the reproducibility of the developed approach (Figure 2B).

### 3.2 Optimization of conditions for quantum dot synthesis by QD4

#### 3.2.1 Optimization of growth conditions of quantum dot bacteria

**Figure 3:**
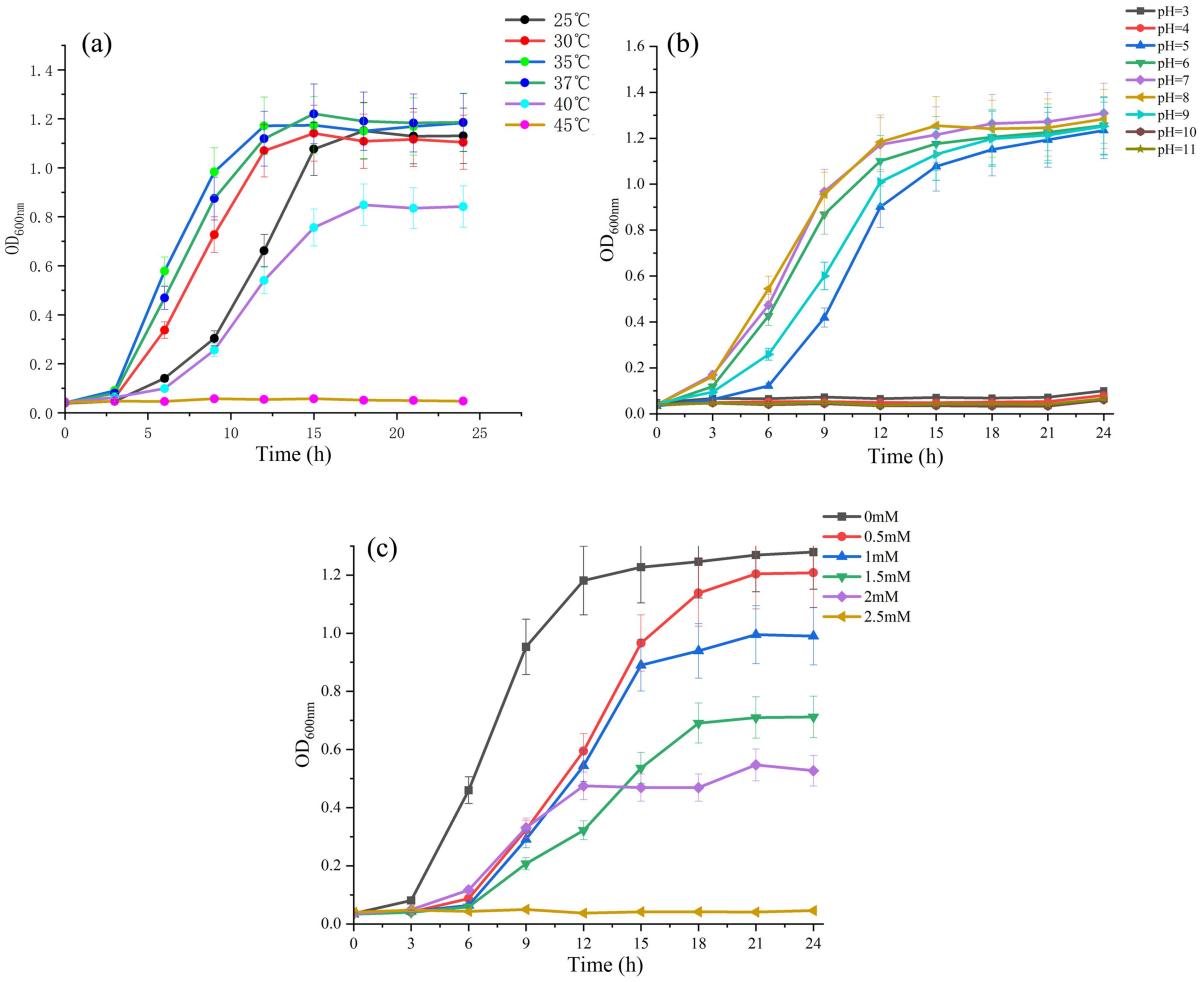
Effect of different culture conditions on the growth of QD4 strain. Growth curves at different **(A)** temperatures, **(B)** pH, and **(C)** cadmium ion concentrations.

The effects of temperature and pH on the growth of the QD4 strain are shown in Figure 3. The strain demonstrates its high degree of environmental adaptability by growing in a broad range of temperatures (25 °С to 40 °С) and pH levels (5 to 9). The best growth temperature of the QD4 strain was achieved at a temperature of 35 °С–37 °С (Figure 3A) and an optimal pH range of 7–8 (Figure 3B). Therefore, 37 °С and pH 7 are selected as optimum temperature and pH for the biosynthesis of quantum dot by QD4.

#### 3.2.2 Selection of optimal cadmium ion concentration

Different amounts of cadmium ions were added to the LB liquid medium with inoculated QD4 bacteria. The optical density at 600 nm (OD_600 nm_) is measured at regular intervals to investigate the bacterial growth status. It is evident from Figure 3C that the bacterium could grow in the culture medium when the concentration of cadmium ion is lower than 2.0 mM. This suggests that the QD4 strain has a relatively high Cd resistance. Considering the toxic effects of Cd^2+^ on microorganisms and the requirement of cadmium ions for the production of quantum dots, 1 mM Cd^2+^ is selected as the optimum cadmium ion concentration for subsequent experiments.

#### 3.2.3 Determination of appropriate sulfur source

The QD4 strain was cultured in the presence of CdCl_2_ and two different sulfur sources: glutathione and L-cysteine. The fluorescence of the culture solution was checked using a UV lamp at 365 nm as a function of time. In the presence of L-cysteine, the yellow fluorescence indicating the formation of CdS quantum dots appears rapidly (approximately 2–4 h), whereas no obvious yellow fluorescence was observed in the culture with glutathione, even after a relatively long time reaction. This implies that glutathione is not an ideal source of sulfur for the biosynthesis of CdS quantum dots by the QD4 strain. Based on the fluorescence comparison, L-cysteine is selected as the optimal sulfur source for QD4.

#### 3.2.4 Determination of optimum induction time

**Figure 4:**
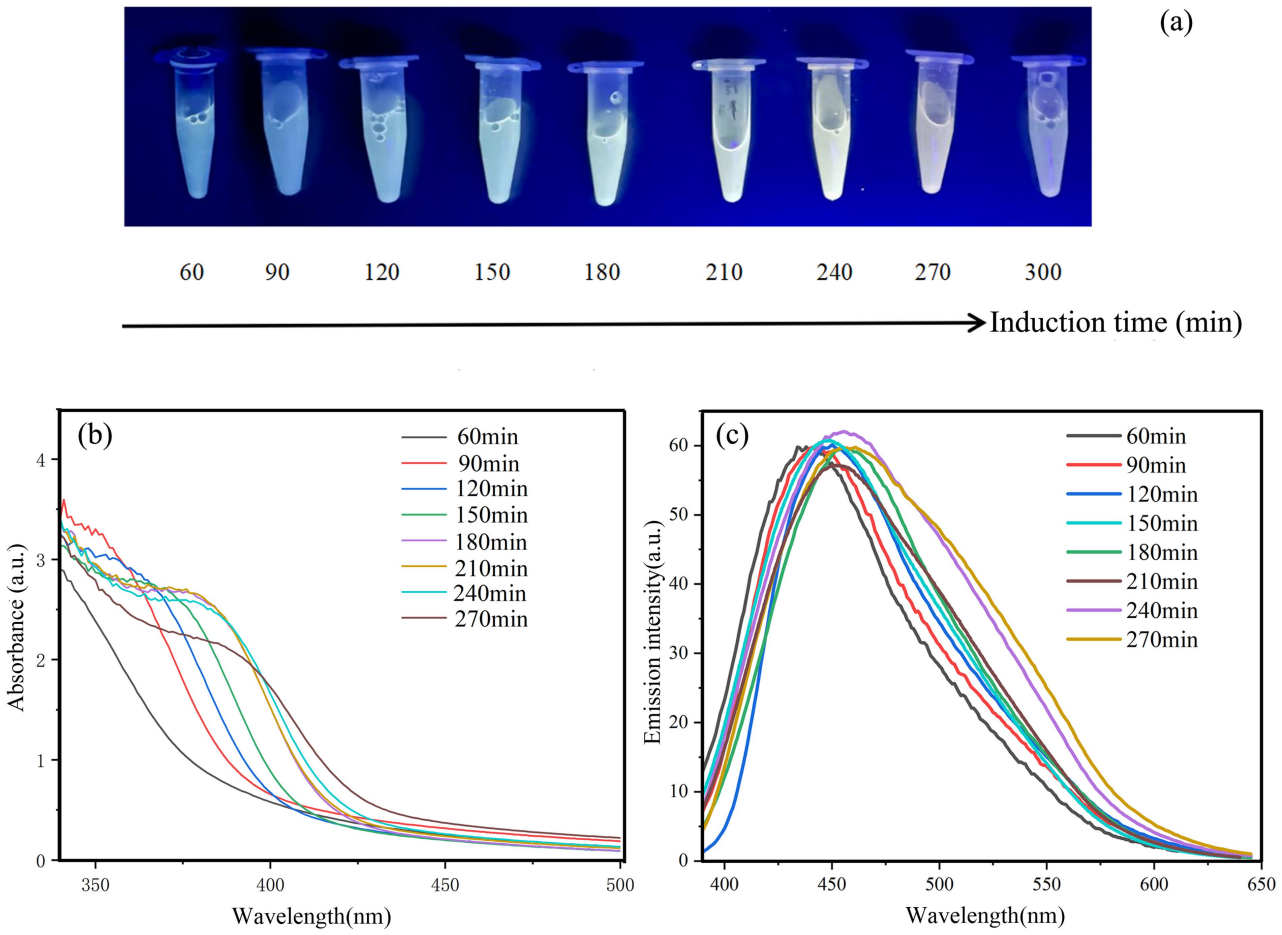
Optical properties of CdS quantum dots by strain QD4 at varying induction times. **(A)** Photographs of the culture supernatant of strain QD4 under UV irradiation (365 nm); **(B)** UV absorption spectra of CdS quantum dot; and **(C)** fluorescence emission spectra of CdS quantum dot, using a 365 nm excitation wavelength.

Using the optimized bacterial culture conditions, the QD4 strain is cultured in the LB liquid medium up to OD_600 nm_ = 1, and then, 1 mM Cd^2+^ and 8 mM L-cysteine are added into the bacterial solution for further reaction. Figure 4A shows the photographs of the reaction solution at various times under UV light. The color and intensity of fluorescence change obviously with time. The bright yellow fluorescence (typical color of CdS) starts to appear after 2 h and disappears after 6 h. The absorption and fluorescence peaks shifted systematically, as the increase of induction time (Figures 4B,C), with the maximum value shifting to a higher wavelength. When the emission wavelength of the samples was scanned at an excitation wavelength of 365 nm, the peak of the 60-min sample was at approximately 440 nm, while the peaks of the 90-, 120-, 150-, 180-, 210-, 240-, and 270-min samples shifted to 443 nm, 445 nm, 449 nm, 453 nm, 455 nm, and 458 nm, respectively. CdS quantum dots can produce different color spectra depending on the particle size. The fluorescence red-shift phenomenon is related to the growth of the CdS quantum dot. According to the size effect of CdS quantum dots, it is estimated that there may be CdS nanoparticles ranging in size from 3 nm to 6 nm in the supernatant. After a longer reaction time, CdS nanoparticles grew larger accompanied by aggregation, leading to the disappearance of fluorescence after 6 h. The relationship between adsorption peak and nanoparticle size is in agreement with the findings of other reports related to L-cysteine-capped CdS quantum dots (Yang et al., 2015).

### 3.3 Quantum dot characterization

**Figure 5:**
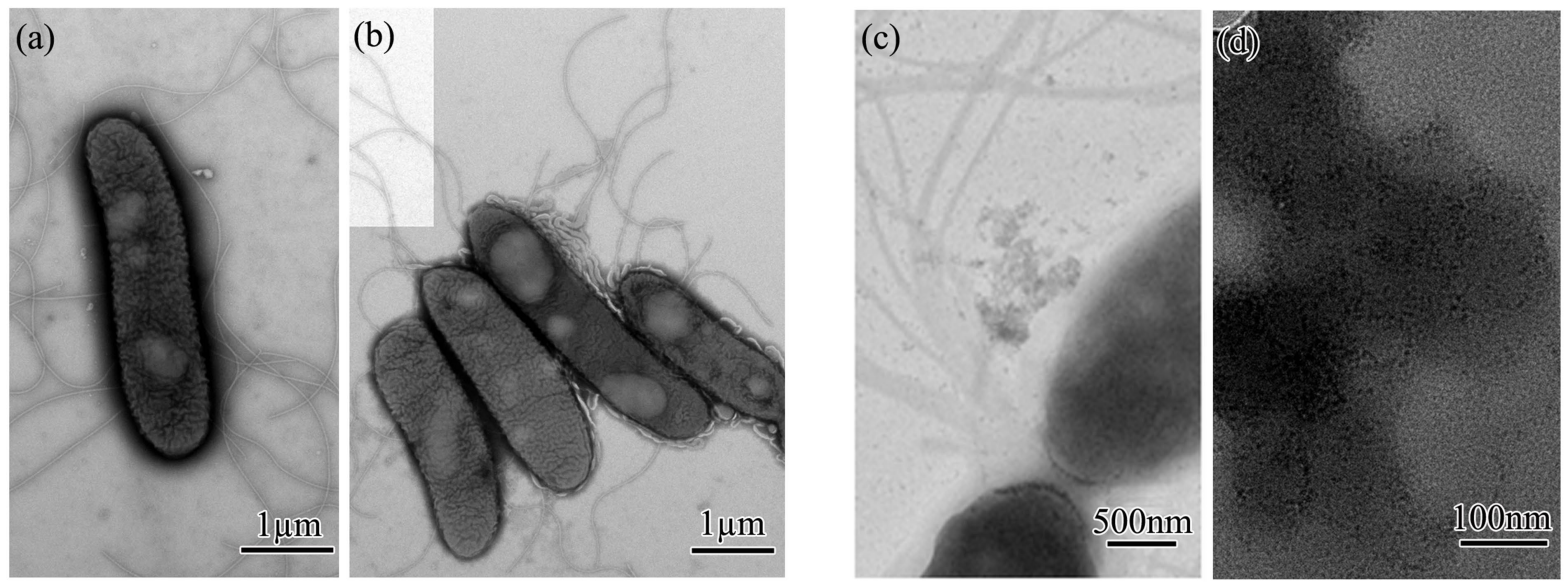
TEM analysis of CdS quantum dot biosynthesized by QD4 bacteria: **(A,B)** Morphology of negatively stained QD4 bacteria. **(C,D)** Morphology of CdS quantum dot biosynthesized extracellularly by QD4 bacteria.

The biosynthesis of CdS quantum dots from strain QD4 is further confirmed through high-resolution transmission electron microscopy (HRTEM). QD4 cells treated with or without Cd^2+^ and L-cysteine were collected and analyzed by TEM. It is evident from TEM images that the cells cultured without any treatment are rod-shaped with several flagella (Figures 5A,B). After 3 h induction, with Cd^2+^ and L-cysteine, the cells are observed to be surrounded by a large number of very fine-grained, crystalline nanoparticles, creating a dark contrast in bright-field image (Figure 5C). These nanoparticles are extracellular and attached to the cell membrane. In addition, nanoparticles are also observed at some distance from the QD4 cells. Figure 5D shows the magnified images of some nanoparticles distributed around the bacterial cells. These results confirm the extracellular synthesis of CdS quantum dots by the bacterium, with an average size being approximately 5 nm.

**Figure 6:**
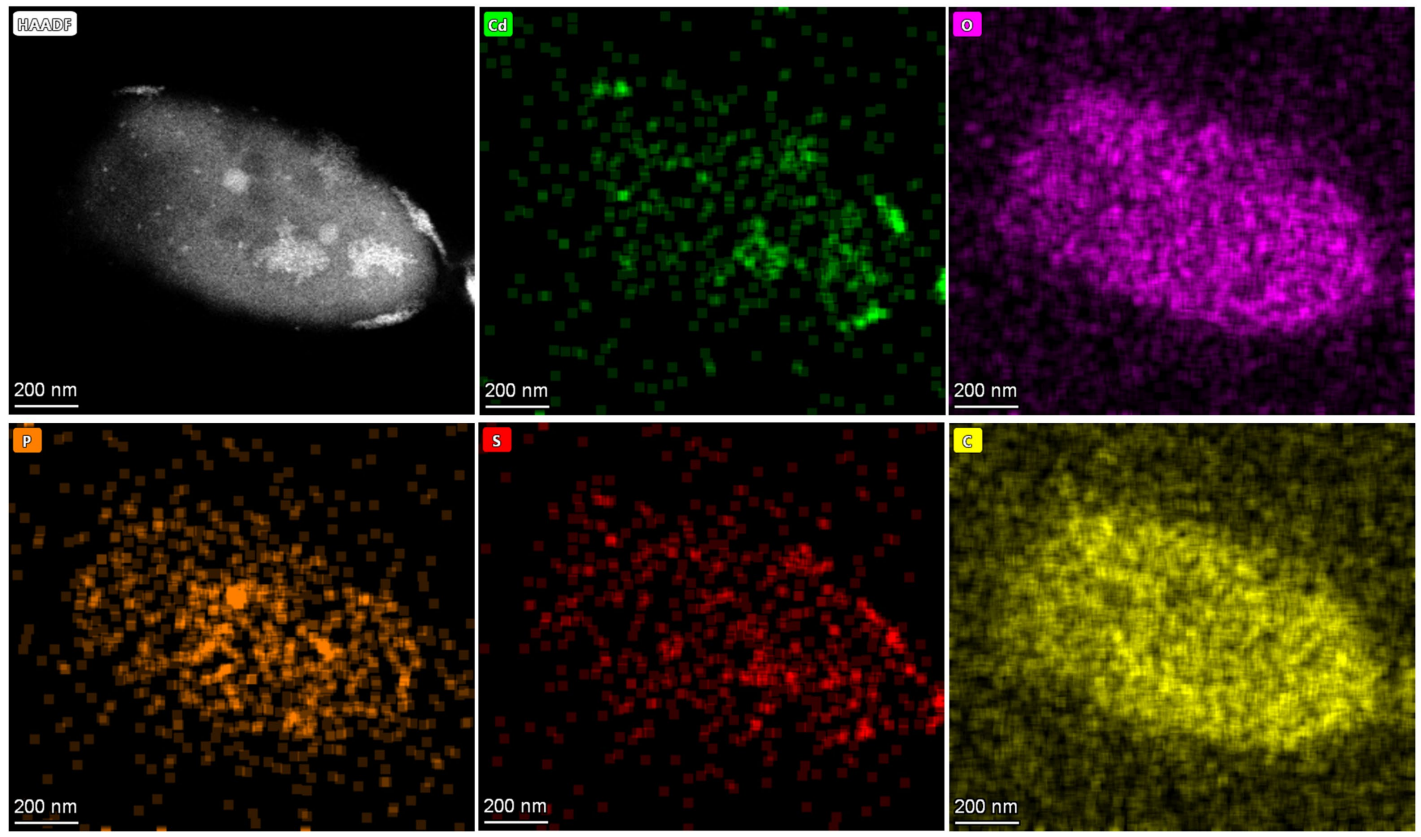
High-angle annular dark field (HAADF) image of one QD4 cell and produced CdS quantum dot, with corresponding EDS maps showing Cd, O, P, S, and C distribution.

QD4 cells with the CdS quantum dots are also analyzed by TEM in scanning transmission (STEM) mode. Figure 6 shows the energy-dispersive X-ray spectrometry (EDS) results of the nanoparticles adhered to one cell. The EDS maps show the clusters of nanoparticles, overlapped with Cd and S elements, implying that these nanoparticles are indeed CdS. Based on the distribution of CdS nanoparticles, it can be concluded that the formation of CdS quantum dots by the QD4 strain occurred both extracellularly and intracellularly.

**Figure 7:**
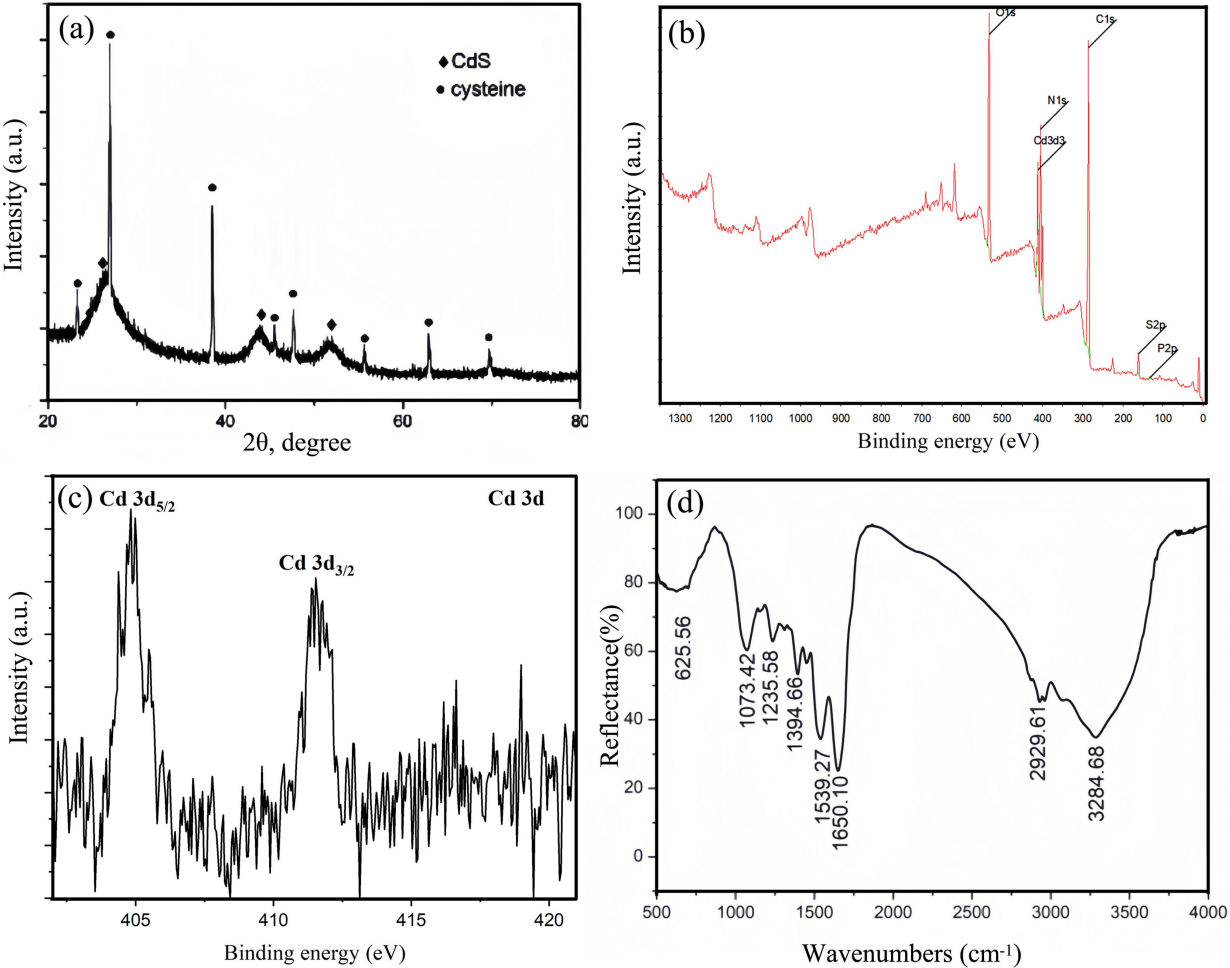
Characterization of precipitated CdS nanoparticles powder after 24 h of growth. **(A)** XRD spectrum; **(B)** XPS survey spectrum; **(C)** XPS spectrum of Cd 3d; and **(D)** FTIR spectrum of CdS nanoparticles.

The CdS quantum dots in the supernatant of QD4 cultures are purified and characterized. The synthesized CdS nanoparticles grew larger and appeared in the bacterial solution as yellow precipitation after a relatively long reaction time (approximately 24 h) with Cd^2+^ and L-cysteine. The yellow precipitation was collected through centrifugation and then washed and dried to obtain a powder form. These powders were divided into three parts for XRD, XPS, and FTIR analysis, respectively. Figure 7A shows the XRD pattern of CdS nanoparticles, with three characteristic peaks (labeled “◆”) corresponding to 111, 220, and 311 planes of cubic CdS (PDF card no. 89–0440), respectively. The broadening of the diffraction peaks of CdS can be attributed to the nanoscopic nature of the nanoparticles and some amorphous tendencies. In addition to the diffraction peaks labeled “◆” (corresponding to the physical phase of CdS), there are also some interferences of diffraction peaks labeled as “●,” which correspond to the crystalline L-cysteine substrate (Figure 7A). This could be because the amount of L-cysteine added for CdS biosynthesis is relatively excessive, while its water solubility is poor, leading to precipitation of excessive and unconsumed L-cysteine. XPS analysis is performed to determine the composition and valence state of compounds. This technique is used to assess the spectra of CdS nanoparticles biosynthesized by QD4. Survey spectra of CdS nanoparticles demonstrate the presence of C 1 s, Cd 3d, N 1 s, O 1 s, P 2p, and S 2p (Figure 7B), suggesting that the main elements of the sample are C, O, N, Cd, S, and so on. This finding is consistent with TEM composition analysis (Figure 6). Cd 3d deconvoluted peaks (Figure 7C) reveal the binding energies of 412 and 405 eV, corresponding to Cd_3/2_ and Cd_5/2_ as reported, which confirmed the presence of CdS compounds (Marusak et al., 2016). FTIR spectra are displayed in Figure 7D. The position of infrared absorption peaks confirms the presence of L-cysteine groups on the surface of CdS quantum dots produced by QD4.

## 4 Discussion

The use of bacteria as cell factories to produce economically and technologically valuable nanoparticles has grown in recent years, providing a safe and green alternative to chemical and physical synthesis processes. It also provides the possibility to produce nanoparticles with novel characteristics and better application potential. In this study, a novel cadmium-resistant bacterium, *Lysinibacillus boronitolerans* QD4, is isolated from seawater. This strain has the ability to efficiently biosynthesize CdS quantum dots using Cd^2+^ and L-cysteine. Based on morphology, physio-biochemistry, and 16S rRNA analysis, it is identified as *Lysinibacillus boronitolerans*. The optimum conditions of quantum dots synthesis using QD4 are found to be 37 °C, pH = 7.0, Cd^2+^ concentration of 1 mM, and L-cysteine as the source of sulfur, with an induction time of 240 min. These optimal conditions are used in further experiments for efficient biosynthesis of CdS quantum dots through QD4.

There are several advantages of the biosynthesis of CdS quantum dots by strain QD4 for industrial applications. First, the strain QD4 is non-pathogenic, which can grow well in LB media at 25 °C–40 °C and 5.0–9.0 pH under aerobic conditions. Compared to the reported anaerobic bacteria used for the biosynthesis of CdS quantum dot (Jang et al., 2015; Qi et al., 2019; Rajput et al., 2021; Chellamuthu et al., 2019), aerobic bacteria are much easier to cultivate and save costs. Meanwhile, QD4 has a fast growth rate. Only after 10 h of cultivation (see Figure 3A), the strain QD4 can achieve a logarithmic growth phase. Therefore, the strain offers the benefits of great environmental adaptation, ease of culture, and non-pathogenicity. Second, the strain QD4 could grow at a Cd^2+^ concentration of up to 2 mM (see Figure 3C), which indicates its outstanding tolerance to the toxic Cd metal. Bacteria such as *Idiomarina* sp. OT37-5b, *Escherichia coli*, and others show a Cd tolerance limit of approximately 0.4 mM (Mi et al., 2011; Ma et al., 2021). One of the ideal bacteria for biosynthesizing CdS quantum dots in previous reports, *Stenotrophomonas maltophilia*, has been shown to tolerate high Cd^2+^ concentrations of more than 1 mM (Pages et al., 2008; Yang et al., 2015). Correspondingly, the high Cd resistance of strain QD4 indicates that the bacterium has very good environmental adaptability and potential for application. Third, the biosynthesis of CdS quantum dots using QD4 only takes a few hours, according to the induction experiment (Figure 4), while many reported bacteria need a relatively long time (approximately 1–5 days) for the biosynthesis of CdS quantum dots (Oliva-Arancibia et al., 2017; Ulloa et al., 2016; Ma et al., 2021; Bruna et al., 2019). The ability of efficient and rapid biosynthesis of CdS quantum dots is a significant advantage of the screened strain QD4. Fourth, the culture supernatant can retain the yellow photoluminescence from CdS quantum dots after the removal of cells through centrifugation. This indicates the extracellular biosynthesis of water-soluble fluorescent particles. Furthermore, TEM analysis (Figures 5, 6) directly shows a larger number of CdS quantum dots distributed extracellularly around the QD4 bacterium cells. It is significant to note that the water-soluble CdS quantum dot produced extracellularly by QD4 is another apparent advantage for industrial application due to the simplified purification of CdS quantum dots. As reported in previous studies, harvesting intracellular nanoparticles requires cell lysis, as well as may also introduce post-production alterations to the nanoparticles. Therefore, extracellular biosynthesis should be considered while designing biosynthesis routes for nanoparticles.

The findings of the study also show that L-cysteine, rather than glutathione, is a better source of sulfur for the biosynthesis of CdS quantum dots by QD4. Moreover, the mechanism for CdS quantum dots biosynthesis using QD4 is supposed to be as follows. One path of CdS quantum dot biosynthesis is through the conversion of L-cysteine to H_2_S catalyzed by cysteine desulfhydrase or homologous enzyme. For example, *Stenotrophomonas maltophilia* used cystathionine *γ*-lyase enzyme to catalyze the biosynthesis of CdS quantum dot (Dunleavy et al., 2016), while *Raoultella* sp. strain X13 was reported to possess the genes that potentially encode cysteine desulfhydrase, as indicated by the presence of up to five open reading frames code related to these enzymes (Xu et al., 2019). This suggests that strain QD4 must contain some enzymes related to cysteine desulfhydrase. In future research, molecular mechanisms of the biosynthesis of CdS quantum dots by strain QD4 need to be investigated for the application of this strain at an industrial scale.

It is important to note that the autofluorescence from the bacterial culture may obstruct detection, especially in the early stage of CdS quantum dot production. The bacterial culture can also show apparent blue fluorescence, in the absence of any additives such as Cd^2+^ or L-cysteine. Therefore, the yellow characteristic fluorescence from CdS quantum dots being observed is very necessary to avoid the interference of spontaneous fluorescence. Successful biosynthesis of CdS quantum dots in the bacterial solution was considered only after the appearance of yellow fluorescence in solution.

In this study, a novel approach is explored for the extracellular biosynthesis of water-soluble CdS quantum dots using a new *Lysinibacillus boronitolerans* QD4 strain. It can serve as an ideal cell factory for environment-friendly biosynthesis of these nanoparticles, which can be further used for industrial applications. It easily grows in simple growth media and can rapidly adapt to environmental changes, showing great prospects as a quantum dot producing “bio-factory.”

## Data Availability

The 16S rDNA sequence has deposited in NCBI (https://www.ncbi.nlm.nih.gov/) under the GenBank accession number PQ881878.
